# Anthropometric Measures and Physical Activity and the Risk of Lung Cancer in Never-Smokers: A Prospective Cohort Study

**DOI:** 10.1371/journal.pone.0070672

**Published:** 2013-08-05

**Authors:** Tram Kim Lam, Steve C. Moore, Louise A. Brinton, Llewellyn Smith, Albert R. Hollenbeck, Gretchen L. Gierach, Neal D. Freedman

**Affiliations:** 1 Genetic Epidemiology Branch, Division of Cancer Epidemiology and Genetics, National Cancer Institute, National Institutes of Health, Rockville, Maryland, United States of America; 2 Nutritional Epidemiology Branch, Division of Cancer Epidemiology and Genetics, National Cancer Institute, National Institutes of Health, Rockville, Maryland, United States of America; 3 Hormonal and Reproductive Epidemiology Branch, Division of Cancer Epidemiology and Genetics, National Cancer Institute, National Institutes of Health, Rockville, Maryland, United States of America; 4 Williams College, Williamstown, Massachusetts, United States of America; 5 Organizational and Tracking Research Department, AARP, Washington, District of Columbia, United States of America; National Taiwan University, Taiwan

## Abstract

Worldwide, lung cancer in never-smokers is ranked the seventh most common cause of cancer death; however, the etiology of lung cancer in never-smokers is unclear. We investigated associations for body mass index (BMI) at various ages, waist circumference, hip circumference, and physical activity with lung cancer in 158,415 never-smokers of the NIH-AARP Diet and Health Study. Multivariable hazard ratios (HR) and 95% confidence intervals (CI) were estimated from Cox proportional hazards models. Over 11 years of follow-up, 532 lung cancer cases occurred. The risk estimate for obese (BMI≥30 kg/m^2^) participants at baseline was 1.21 (95%CI = 0.95–1.53) relative to those with a normal BMI between 18.5≤BMI<25.0. Overweight (25.0≤BMI<30.0) at age 18 (HR_overweight-vs-normal_ = 1.51;95%CI = 1.01–2.26) and time spent sitting (HR_≥3 hrs-vs-<3 hrs_ = 1.32;95%CI = 1.00–1.73) was each associated with lung cancer after adjustment for baseline BMI, as was waist (HR_Q4-vs-Q1_ = 1.75;95%CI = 1.09–2.79) and hip circumference (HR_Q4-vs-Q1_ = 0.62;95%CI = 0.39–0.99), after mutual adjustment for each other and baseline BMI. No associations were observed for vigorous activity or television watching. In summary, using a large prospective cohort study, we found no evidence that BMI at baseline or middle age was associated with decreased lung cancer risk in never smokers. If anything, we observed some evidence for positive associations with a larger BMI or waist circumference.

## Introduction

Lung cancer is perceived as a smoker’s disease; nevertheless, approximately 10–15% of lung cancers occur in never-smokers [1], [2]. Worldwide, lung cancer in never-smokers is ranked the seventh most common cause of cancer death [3], [4] and in the United States, it has been estimated to cause between16,000 to 24,000 deaths per year [2]. Several risk factors, including secondhand smoke, indoor radon, household coal smoke, prior lung disease, and particular genetic susceptibility loci [5] have been associated with lung cancer in never-smokers. Despite these factors, the etiology of lung cancer in never-smokers remains unclear [3], [6]. Sisti and Boffetta reviewed the literature and reported the burden of lung cancer in never-smokers attributable to identified risk factors (associated population attributable fractions ranged from 0.40% to 19.3%) [7]. In their conclusion, the authors commented on the scarcity of epidemiologic studies of lung cancer in never-smokers, particularly among Western countries, and recommended that additional epidemiologic studies are warranted [7].

Body size and shape may be related to lung cancer. Studies have suggested that a high body mass index (BMI–defined as the weight in kilograms divided by the square of height in meters) is associated with a lower risk of lung cancer. In a systematic review of 13 prospective cohort studies, obesity (a BMI of 30.0 kg/m^2^ or greater) was associated a 20–24% with lower risk of lung cancer (HR_pooled_ = 0.76; 95% CI = 0.70–0.83 in men; HR_pooled_ = 0.80; 95% CI = 0.66–0.97 in women) [8]. In contrast, lung cancer risk has been positively associated with waist circumference [9], [10] and inversely associated with physical activity, respectively, in smokers [11], [12]. However, whether such findings apply to lung cancer in never-smokers is unclear, as most previous prospective studies [8], [12], [13] have had limited numbers of lung cancer cases in never-smokers. Prior studies have also not evaluated the relation between sedentary behaviors and risk among never-smokers. Therefore, we examined the association of obesity, using multiple measures of body shape, physical activity, and sedentary behaviors with lung cancer risk in never-smokers in the National Institute of Health (NIH)-AARP Diet and Health cohort. The large size and extended follow-up of this cohort yielded more than 500 cases of incident lung cancer in never-smokers, substantially more than in previous studies.

## Materials and Methods

### Ethics Statement

The study was approved by a Special Studies Institutional Review Board of the U.S. National Cancer Institute.

### Study Population

The NIH-AARP Diet and Health Study has been described previously [14]. Briefly, the study recruited men and women by mailing questionnaires to 3.5 million AARP members aged 50–71 years old from six US states (CA, FL, LA, NJ, NC, and PA) and 2 metropolitan areas (Atlanta, GA and Detroit, MI) between 1995–1996. A second risk factor questionnaire requesting information on additional risk factors, including waist and hip circumference, weight and height at various points in adulthood, was completed by 337,074 baseline participants six months after the baseline questionnaire.

### Cohort Follow-up

Cohort members were followed annually for address changes and vital status from baseline through December 31, 2006. Address changes were identified through linkage to the U.S. Postal Service’s National Change of Address database, other update services, and direct participants’ notifications. Vital status was updated through linkage to the Social Security Administration Death Master File and verified by the National Death Index.

### Case Ascertainment

We identified cancer cases through probabilistic linkage with 10 state cancer registry databases that included the 8 original states and 3 additional states (Arizona, Nevada, and Texas) that some participants moved to during follow-up.

Dates of diagnosis and tumor characteristics were obtained from the cancer registries. Using histologic codes from the *International Classification of Disease for Oncology* (ICD-O, third edition) [15] all primary incident cancers of the bronchus and lung (ICD 34.0–34.9) were considered for the present analysis. By histologic code, lung carcinomas included small cell (8002, 8041, 8042, 8043, 8044, 8045), adenocarcinoma (bronchoalveolar: 8250, 8251, 8252, 8253, 8254, and other: 8140, 8255, 8260, 8310, 8323, 8480, 8481, 8490, 8550, and 8574), squamous cell (8050, 8070, 8071, 8072, 8073, 8074, and 8075), undifferentiated/large cell (8012, 8020, 8021, 8022, 8031, and 8032), and other not otherwise specified (NOS) carcinoma (8010, 8011, 8033, 8046, and 8560).

### Analytical Cohort/subcohort

Of the 566,401 individuals who returned the baseline questionnaire, we excluded those who had previous cancer at baseline (n = 51,234), proxy respondents (n = 15,760), and those with extreme values (more than two times inter-quartile ranges from the median) of box-cox log transformed total energy intake (n = 4,417), and ever smokers of cigarettes, pipes, or cigars (n = 336,575). Our analytic cohort included 158,415 never-smokers (male = 70,721 and female = 87,694).

For exposures assessed on the second risk factor questionnaire, we created an analytical subcohort restricted to 100,226 never-smokers (male = 43,511 and female = 56,715) who responded to the risk factor questionnaire, excluding proxies ( = 971).

### Exposure Assessment

We derived anthropometric variables (baseline weight and height) from the baseline questionnaire and the second risk factor questionnaire (waist circumference, hip circumference, weight and height at 18, 35, and 50 years). BMI at different ages (18, 35, 50, and at baseline) was computed as measured weight (kg) divided by the square of height (m^2^). With the exception of BMI at 18, for which we used height at 18 years, we used baseline height in the calculation of BMI. We categorized all BMI variables according to World Health Organization (WHO) definitions [16] (<18.5 kg/m^2^, 18.5-<25.0, 25.0-<30.0, and 30+). Participants were asked to measure their waist circumference with a tape measure one inch above the navel and the largest spot for their hip circumference while standing. They were asked to report values to the nearest quarter inch. Those without a tape measure were asked to leave a blank response (waist_missing_ = 24%; hip_missing_ = 27%). Compared to the total analytical cohort, these individuals did not significantly differ in age and education and were excluded from the analyses. Waist-hip-ratio was calculated as waist circumference divided by hip circumference.

We categorized weight, height, waist circumference, hip circumference, and waist-hip-ratio by sex-specific quartiles based on the distribution of the entire the analytical cohort or subcohort from which the variables were derived.

Information on physical activity at baseline was obtained from two questions: (1) physical activity at work and (2) vigorous activity, defined as the frequency each week spent at activities that lasted 20 minutes or more and caused either increases in breathing or heart rate or working up a sweat. We classified activities at work into 4 categories: (1) lift and carry loads; (2) walking a lot; (3) mostly sitting with a fair amount of walking; and (4) all day sitting. Categories of vigorous activity were: <1 time per week, 1–2 times per week, 3–4 times per week, and 5+ times per week.

Information on sedentary behaviors was based on two questions from the risk factor questionnaire: (1) time spent watching TV or videos during a typical 24-hour period over the past 12 months; and (2) number of hours spent sitting during a typical 24-hour period over the past 12 months. We classified TV watching or videos and hours spent sitting to the following categories: <3, 3–4, and ≥5 hours per day. For hours spent sitting, we also dichotomized categories into <3 and ≥3 hours per day.

### Statistical Analysis

We used multivariable Cox proportional hazards regression models [17] with person-time as the time scale to estimate sex-specific HRs and 95% CIs. For the analytical cohort and subcohort, person-years of follow-up time were calculated from the date of the questionnaire, either baseline or risk factor as appropriate, until the date of cancer diagnosis, death, movement out of the registry areas, or end of follow-up (December 31^st^, 2006), whichever occurred first. For BMI, waist circumference, hip circumference, and waist-hip-ratio, linear trend was tested by assigning a median value for each category and included them in the statistical model. We considered the other categorical variables of interest (TV watching, sedentary behavior, and physical activity at work and baseline) as an ordinal variable and tested for linear trend. The coefficient for each was evaluated using a Wald test.

All models were adjusted for age, ethnicity, education (<less than high school, 12 years, some post-high school training, completed college, and completed graduate school), alcohol intake (0, >0–1, >1–3, and >3 drinks/day), vigorous physical activity, physical activity at work, and total energy (continuous). As sensitivity analyses, we additionally adjusted analyses of BMI at 18, 35, and 50 years of age and of sedentary behaviors for baseline BMI. Models for waist and hip circumference were presented individually, mutually adjusted for each other, and for baseline BMI. Further adjustment for intake of fruits and vegetables (continuous) or, in women, menopausal hormone therapy (MHT, defined as ever or never use) did not materially alter the results and thus were not included in the final models. We examined risk estimates in men and women separately, but found similar associations in both. Therefore, we present combined risk estimates in the present report. We also performed lag analyses by excluding events occurring during the first three years of follow-up. The proportional hazards assumption was verified using time interaction models.

All analyses were performed using SAS (SAS Institute, Cary, NC). We interpreted *P*<0.05 and/or 95% CIs that excluded 1 as statistically significant and all tests were two-sided.

## Results

Approximately 37% of the cohort had normal BMI (between 18.5-<25 kg/m^2^) at baseline (Table1). Less than 4% of the cohort had a BMI <18.5 kg/m^2^, 40% of the cohort was overweight (BMI between 25-<30 kg/m^2^), and 20% was obese (BMI ≥30 kg/m^2^). For both males and females, obese participants tended to be younger, had fewer years of education, reported less physical activity, and consumed less fruits and vegetables than non-obese subjects. More obese men reported the heaviest alcohol consumption (3+ drinks/day) whereas obese women reported less heavy drinking than their non-obese counterparts. As expected, baseline BMI was correlated with BMI at 50 years-old, waist circumference, and current weight (Table S1).

**Table 1:** Selected characteristics by BMI categories (kg/m2) in the NIH-AARP Diet and Health Study (1995–2006).

Variable	Whole cohort	Male (n = 70,721)				Female (n = 87,694)			
	(n = 158,415)	<18.5	18.5–<25.0	25.0–<30.0	30+	<18.5	18.5–<25.0	25.0–<30.0	30+
Number (%)	158,415	1,863 (2.6)	23,476 (33.2)	33,124 (46.8)	12,258 (17.3)	4,039 (4.6)	35,327 (40.2)	27,837 (31.7)	20,491 (23.3)
Age, mean (SD)	61.8 (5.3)	62.2 (5.2)	61.9 (5.4)	61.6 (5.3)	60.85 (5.3)	62.7	61.9 (5.4)	62.2 (5.3)	61.6 (5.3)
Education (%)^1^									
Less than high school	4.7	7.3	2.3	3.6	4.6	11.3	2.8	5.8	7.5
12 years (completed high school)	21.2	16.3	10.2	13.5	16.6	30.4	24.7	29.5	30.2
Some post-high school training	30.3	26.1	22.6	26.5	30.2	28.9	33.3	34.2	35.3
Completed college	19.4	21.0	25.8	24.2	21.6	15.0	18.0	14.6	12.6
Completed graduate school	24.5	29.3	39.2	32.2	27.1	14.1	20.0	15.8	14.1
Vigorous physical activity (%)^1^									
Never	4.1	5.2	2.1	2.3	4.2	7.5	2.7	4.8	8.2
Rarely	12.9	12.0	7.0	9.2	15.3	15.9	11.3	15.5	23.1
1–3 times/month	13.2	12.8	9.8	12.6	16.9	12.7	11.6	14.1	17.1
1–2 times/week	21.9	19.0	19.8	23.6	24.7	18.8	20.7	22.7	21.3
3–4 times/week	27.4	26.8	32.3	29.8	23.1	22.9	29.6	26.3	19.2
5 or more times/week	19.4	22.1	28.3	21.9	14.9	18.6	21.7	15.2	9.5
Alcohol intake (%)^1^									
0 drink/day	31.2	30.9	24.6	25.2	30.0	44.8	29.5	35.2	43.9
>0 to 1 drink/day	56.1	52.3	55.3	55.1	54.2	48.2	60.2	57.6	52.0
>1 to 3 drinks/day	9.9	12.4	15.9	14.5	10.4	6.0	9.0	5.9	3.0
>3 drinks/day	2.8	4.4	4.2	5.2	5.4	0.9	1.2	1.2	0.9
Total daily calories*,a	1,615	1,810	1,790	1,824	1,927	1,451	1,425	1,457	1,533
Total fruit intake*,b	1.9	1.9	1.6	1.5	1.4	1.9	1.9	1.8	1.6
Total vegetable intake*,b	2.3	2.2	1.9	1.9	1.9	2.2	2.2	2.2	2.1

* Median intake; ^a^kcal/d; ^b^servings/1,000 kcal.

1 Numbers/percentage might not add to 100% due to missing data.

During 1,578,092.66 person-years of follow-up (mean follow-up = 9.96 years; SD = 2.04), we identified 532 lung cancer cases (n = 198 males and n = 334 females) among never-smokers. Those who responded to the risk factor questionnaire contributed a total of 947,382.96 person-years of follow up (mean follow-up = 9.45 years; SD 1.89) with 332 lung cancer cases. As expected, the majority of cases had adenocarcinoma histology (54%) followed by other non-small cell subtypes and undifferentiated lung cancer. A small fraction (5%) of the cases were identified as small cell lung cancer.

Table 2 presents the multivariable-adjusted hazard ratio and corresponding confidence intervals for the association between BMI at various age points with lung cancer. The risk estimate for obesity at baseline with lung cancer was 1.21 (95% CI = 0.95–1.53; *p*-trend = 0.21), relative to having a BMI in the normal range. Results for BMI at age 50 were similar (HR_unadjusted-for-BMI-at- baseline_ = 1.25; 95% CI: 0.88–1.77; *p*-trend = 0.71) to those for BMI at study baseline. Our data showed no association with obesity at age 35 and lung cancer risk. Only three individuals reported being obese at age 18; the associated hazard ratio was 0.86 (95% CI = 0.27–2.69; *p*-trend = 0.72). For this age group, there was a suggestion that individuals who were overweight at 18 years had a 46% (95% CI = 0.99–2.17) increased risk of lung cancer compared to normal weight individuals. Additional adjustment for baseline BMI strengthened the risk estimates (HR = 1.51; 95% CI = 1.01–2.26).

**Table 2:** Hazard ratios (HR)and 95% confidence intervals (CI) for body mass index (BMI) at various ages and lung cancer risk among never smokers, NIH-AARP Diet and Health Study (1995–2006).

	<18.5	18.5–<25.0	25.0–<30.0	30+	*p*-trend
**BMI^§^, baseline**					
Case count	23	194	192	123	
HR^1^ (95% CI)	1.57 (0.77–3.19)	1.00 (ref)	1.00 (0.81–1.22)	1.21 (0.95–1.53)	0.21
**BMI^§^, at 50 years**					
Case count	7	167	91	45	
HR^1^ (95% CI)	1.41 (0.62–3.19)	1.00 (ref)	0.83 (0.64–1.08)	1.25 (0.88–1.77)	0.71
HR^1,2^ (95% CI)	1.37 (0.78–3.34)	1.00 (ref)	0.90 (0.66–1.21)	1.50 (0.92–2.42)	0.62
**BMI^§^, at 35 years**					
Case count	10	217	69	15	
HR^1^ (95% CI)	1.02 (0.52–2.00)	1.00 (ref)	1.04 (0.79–1.39)	1.08 (0.63–1.84)	0.70
HR^1,2^ (95% CI)	1.01 (0.51–1.98)	1.00 (ref)	1.09 (0.80–1.48)	1.18 (0.66–2.13)	0.60
**BMI^§^, at 18 years**					0.72
Case count	47	209	29	3	
HR^1^ (95% CI)	1.02 (0.74–1.41)	1.00 (ref)	1.46 (0.99–2.17)	0.86 (0.27–2.69)	0.72
HR^1,2^ (95% CI)	0.94 (0.67–1.32)	1.00 (ref)	1.51 (1.01–2.26)	0.91 (0.29–2.90)	0.64

NOTE: ^§^BMI = Body mass index (kg/m^2^) as defined in **Table 1**.

1 Adjusted for age, education, ethnicity, alcohol consumption, vigorous physical activity, physical activity at work, total caloric intake.

2 Additionally adjusted for BMI (continuous) at baseline.

Waist circumference, adjusted for hip circumference, was associated with a 60% increase in lung cancer risk (HR_Q4-vs-Q1_ = 1.60; 95% CI = 1.01–2.52; *p*-trend = 0.12, Table 3), which became stronger after additional adjustment for body mass index at baseline (1.75, 95% CI = 1.09–2.79; *p*-trend = 0.07). Conversely, we observed an inverse association with hip circumference (HR_Q4-vs-Q1_ = 0.61; 95% CI = 0.38–0.97; *p*-trend = 0.12), adjusted for waist circumference, which persisted after additional adjustment for baseline BMI (0.62; 0.39–0.99; *p*-trend = 0.17). Trends across categories of each exposure did not reach statistical significance. We observed no evidence for an association with waist-hip-ratio, either before or after adjustment for baseline BMI. Likewise, we observed no associations with weight and height at baseline (data not shown).

**Table 3:** Hazard ratios (HR) and 95% confidence intervals (CI) for waist/hip ratio, waist circumference, and hip circumference in relation to lung cancer risk among never smokers, NIH-AARP Diet and Health Study (1995–2006).

Anthropometric measures	Quartile*				
	Q1*	Q2*	Q3*	Q4*	*p*-trend
**Waist circumference**					
Case count	63	59	69	64	
HR^1^ (95% CI)	1.00 (ref)	1.04 (0.76–1.41)	1.05 (0.78–1.41)	1.09 (0.80–1.47)	0.94
HR^1,3^ (95% CI)	1.00 (ref)	1.26 (0.88–1.79)	1.41 (0.96–2.07)	1.60 (1.01–2.52)	0.12
HR^1,2,3^(95% CI)	1.00 (ref)	1.25 (0.87–1.79)	1.49 (1.02–2.19)	1.75 (1.09–2.79)	0.07
**Hip circumference**					
Case count	60	62	62	57	
HR^1^ (95% CI)	1.00 (ref)	0.90 (0.66–1.21)	0.87 (0.65–1.18)	0.88 (0.65–1.21)	0.84
HR^1,3^ (95% CI)	1.00 (ref)	0.76 (0.54–1.08)	0.66 (0.45–0.98)	0.61 (0.38–0.97)	0.12
HR^1,2,3^(95% CI)	1.00 (ref)	0.76 (0.54–1.09)	0.64 (0.43–0.95)	0.62 (0.39–0.99)	0.17
**Waist-Hip-Ratio**					
Case count	51	60	66	64	
HR^1^ (95% CI)	1.00 (ref)	1.15 (0.80–1.67)	1.17 (0.81–1.69)	1.14 (0.78–1.65)	0.51
HR^1,2^ (95% CI)	1.00 (ref)	1.14 (0.78–1.66)	1.22 (0.84–1.78)	1.22 (0.83–1.81)	0.26

1 Adjusted for age, education, ethnicity, alcohol consumption, vigorous physical activity, physical activity at work, total caloric intake.

2 Additionally adjusted for BMI (continuous) at baseline.

3 Model with waist, adjusted for hip; Model with hip, adjusted for waist.

* Sex-specific quartiles based on the entire subcohort (respondents of risk factor questionnaire).

Table 4 presents the results for analyses investigating the association between sedentary behaviors and physical activity with risk of lung cancer. Risk estimates for sitting 3–4 hours or 5+ hours per day were above one, and although there was no apparent trend, there was a borderline significant 32% (95% CI = 1.00–1.73) increase in lung cancer risk relative to those who sat less than 3 hours per day after combining these two highest categories. Although point estimates for those sitting all day at work, relative to those lifting and carrying heavy loads, were above one, they were far from statistically significant (HR = 1.21, 95% CI = 0.84–1.76). We observed no evidence for associations between time spent watching television or participating in vigorous physical activities with lung cancer.

**Table 4:** Hazard ratios (HR)and 95% confidence intervals (CI) between sedentary behaviors and physical activity and lung cancer risk among never smokers, NIH-AARP Diet and Health Study (1995–2006).

	Case count	HR	95% CI	*p*-trend
**Sitting** 1				
<3 hours	47	1.00	ref	
3–4 hours	70	1.36	1.00–1.85	0.23
5+ hours	89	1.28	0.96–1.72	
**TV watching** 1				
<3 hours	66	1.00	ref	
3–4 hours	94	1.16	0.91–1.48	
5+ hours	45	1.06	0.77–1.46	0.53
**Physical activity at work** 1				
Lift and carry heavy loads	76	1.00	ref	
Walking a lot	131	0.98	0.78–1.23	
Mostly sitting/walking a bit	88	0.98	0.77–1.24	
All day sitting	25	1.21	0.84–1.76	0.43
**Vigorous activity** 2				
5+ times per week	56	1.00	ref	
3–4 times per week	87	1.16	0.90–1.51	
1–2 times per week	119	1.13	0.87–1.45	
<1 time per week	65	1.06	0.78–1.43	0.75

1 Adjusted for age, current body mass index, education, ethnicity, vigorous activity, alcohol consumption, total caloric intake.

2 Adjusted for age, current body mass index, education, ethnicity, alcohol consumption, total caloric intake.

Within each stratum, the category of most active subjects served as the reference group.

When we excluded cases (n_cohort_ = 106; n_subcohort_ = 89) occurring during the first three years of follow-up, the significant positive association for individuals who were overweight at 18 years old became stronger (HR_overweight-vs-normal = _1.77; 95% CI = 1.13–2.77) compared to normal weight individuals. For all other investigated factors, the risk estimates were in the same direction with widened confidence intervals. Associations also were similar in analyses restricted to cases with adenocarcinoma histology (data not shown).

## Discussion

We prospectively investigated the relationship between adiposity-related measures with lung cancer incidence in a large cohort of never-smokers. In contrast to previously reported results from prospective cohort studies of smokers, we observed no evidence for an inverse association between adulthood/middle age obesity and subsequent lung cancer risk. If anything, an obese BMI at baseline was positively associated with lung cancer, although this association was not statistically significant. We also observed some evidence that having an overweight BMI at age 18 was positively associated with lung cancer risk. Positive associations were observed with higher waist circumference and more time spent sitting; whereas hip circumference appeared to be inversely associated with lung cancer. No statistically significant associations were observed with waist-hip-ratio or vigorous physical activity.

Our finding of no association between BMI and incidence of lung cancer corroborated findings from a pooled analyses of five prospective cohort studies in never-smokers (RR_overall_ = 0.91; 95% CI = 0.76–1.10) [8]. The studies included in the meta-analysis had modest case numbers, with the largest being the Million Women Study involving 269 lung cases [18]. A more recent publication from the Agricultural Health Study cohort [13] also observed no association, but had only 51 cases. Together, these prospective results suggest that BMI at middle age is not associated with lung cancer in never-smokers.

Our observation of an association between being overweight in early adulthood and subsequent lung cancer is interesting; however, we know of no other prospective cohort study that has published results examining this relationship in never-smokers. In smokers, the Harvard Alumni Health Study previously observed an association between BMI in early adulthood (∼18 years) and lung cancer mortality (HR_per 2.56 kg/m_^2^ = 1.24; 95% CI = 1.10–1.40) [19]. It is difficult to investigate the relationship between obesity and lung cancer in this early adulthood group as few individuals reported being obese or overweight at this age, our findings coupled with the Harvard’s results, may warrant further inquiries into this relationship.

In mutually adjusted models, we observed evidence that high waist circumference was associated with a 75% increase in lung cancer risk, whereas high hip circumference was inversely associated with a 38% reduced risk. Only a few studies have examined associations between waist circumference and lung cancer. Results from the only two previous cohort studies of never-smokers were inconsistent [9], [10], although several studies observed positive associations among smokers.

Although limited data is available for lung cancer, the contrasting associations observed for waist and hip circumference has been previously reported for both cardiovascular disease and all-cause mortality [20]. In cross-sectional studies, hip circumference has been inversely associated with blood glucose, blood pressure, and lipid levels, whereas waist circumference has been positively associated with each of these measures [21]. Several explanations for these differences have been posited, including that hip circumference reflects visceral fat, whereas waist circumference may reflect subcutaneous gluteofemoral body fat and lean muscle mass [22]–[24]. Growing evidence from *in vitro* and *in vivo* studies suggests that visceral and subcutaneous adipocytes are distinct with respect to several properties, including lyposis, fatty acid storage, gene expression, adiponectin secretion levels, and insulin action and signaling [23]. In any case, our findings of apparently distinct associations for waist and hip circumference with lung cancer in never-smokers merit replication and possibly suggest a number of future research directions.

As increasing evidence suggests that waist and hip circumferences may measure different aspects of body composition and fat distribution, the assumption that high waist-hip-ratio, commonly used in population studies, is an indicator of abundant abdominal fat relative to low gluteal subcutaneous fat has been challenged [25]. Investigators have argued that waist-hip-ratio is a poor measurement of visceral fat, with a difficult interpretation [21]. In our study, we observed non-significant positive association with waist-hip-ratio.

With respect to physical activity and sedentary behaviors, our data suggest that time spent sitting may be positively associated with lung cancer risk, although of borderline statistical significance. In contrast, there was no evidence that vigorous activity affected risk in our study. Little other data on sedentary behavior in lung cancer are available. Conversely, the body of evidence on vigorous physical/recreation activity and lung cancer risk in ever smokers is more extensive. The majority of the studies have found increased physical activity to be associated with decreased lung cancer risk in ever smokers, although, as in the present study, associations in never-smokers have tended to the null [11], [12], [26]. The inverse association observed in prior reports might be due to inadequate control for tobacco smoking [11].

Contrary to studies in smokers, our data showed no evidence of inverse associations with higher BMI and increased physical activity. Why might results for BMI and physical activity in adulthood differ between ever and never-smokers? One explanation is that lung cancer in never-smokers has a unique etiology [3], [6]. Supporting evidence for this hypothesis includes the disproportionately higher proportion of adenocarcinoma and *EGFR* mutations in lung tumors among never-smokers, along with additional clinical and pathological differences between the two tumors [3]. Indeed, the proportion of adenocarcinomas was substantially higher in never-smokers than smokers in our cohort [27]. Differences in the proportion of cancers with adenocarcinoma histology by smoking status does not itself seem to explain differences for BMI, as a recent analysis observed inverse associations between baseline BMI and lung adenocarcinoma among smokers of our cohort [28]. The etiology of never smoking lung cancer remains unclear, with few published studies. Our investigation with respect to multiple anthropometric measures and lung cancer risk in never smokers is among the first such studies.

Another possible explanation for the difference between ever and never-smokers is that restriction to never-smokers eliminates residual confounding by cigarette smoking. Prior studies adjusted for self-reported cigarette smoking but this is an imperfect assessment of overall smoking history. Accordingly, even statistical models that adjust for smoking status cannot completely eliminate confounding by cigarette smoking. Body weight and physical activity level are each associated with cigarette smoking and cigarette smoking is far and away the predominant risk factor for lung cancer. Therefore residual confounding must be considered an important potential reason for why previous studies observed associations.

A notable strength of the study is that it is the largest prospective study of never smoking lung cancer to date. This provides greater statistical power and allows for more precise estimates of effects. The study’s size also permitted us to examine the relationships by sex as the prevalence of never smoking lung cancer is higher in women. Nevertheless, despite being the largest study to date on BMI and anthropometric measures, our sample size is modest and adequate power remains an issue. Our exposures of interest were derived or assessed from self-report questionnaires, which may be vulnerable to reporting bias. Possible misclassification due to measurement errors is most likely be non-differential as participants at baseline would have been unaware that they would subsequently develop lung cancer. Thus, any inaccuracies in recall would be expected to bias the results towards the null. Furthermore, validation studies have shown high correlations for anthropometric measures between self-reported measurements and those made by a trained nurse [29]. Likewise, similar high correlations and accuracy (r>0.80) have been shown for height and weight [30]. There is also reasonable validation and reproducibility of the instruments used to assess physical activity similar to ours [31]. We additionally lacked information on second hand smoking and radon exposure, which are known risk factors for lung cancer in never-smokers and it is possible that participants exposed to second-hand smoke may have higher BMI [32]. Lastly, in the present study we investigated several exposures and their respective association to lung cancer risk. While our findings contribute to a better understanding of the etiology of lung cancer in never smokers, the results should be interpreted with caution as our findings may be due to chance due to multiple comparisons.

In summary, using a large prospective cohort study, we found no evidence that BMI at baseline or middle age was associated with decreased lung cancer risk in never smokers. If anything, we observed some evidence for positive associations with a larger BMI or waist circumference. We also observed a borderline significant inverse association with hip circumference, possibly implicating subcutaneous gluteofemoral fat or lean muscle mass. Larger prospective cohort studies in never-smokers are needed to replicate these findings.

## Supporting Information

Table S1 **Pearson correlations between anthropometric measures in the NIH-AARP Diet and Health Study.**
(DOCX)
