# Supplementary material for: Anthropometric Measures and Physical Activity and the Risk of Lung Cancer in Never-Smokers: A Prospective Cohort Study
Source: PLoS One. 2013 Aug 5;8(8):e70672. doi: 10.1371/journal.pone.0070672 (PMC3734257; doi:10.1371/journal.pone.0070672)
Supplement: Supplementary file 1 [file pone.0070672.s001.docx]

Table S1: Pearson correlations between anthropometric measures in the NIH-AARP Diet and Health Study

Variables	BMI, baseline	BMI, at 18	BMI, at 35	BMI, at 50	Waist circumference	Hip circumference	Weight	Heigh
**Male**								
BMI, baseline	-	0.38	0.63	0.82	0.77	0.67	0.86	-0.08
BMI, at 18	0.38	-	0.62	0.46	0.25	0.25	0.33	-0.02
BMI, at 35	0.64	0.62	-	0.76	0.46	0.42	0.52	-0.08
BMI, at 50	0.82	0.46	0.76	-	0.64	0.56	0.69	-0.08
Waist	0.77	0.25	0.46	0.64	-	0.76	0.79	0.20
Hip	0.67	0.25	0.41	0.56	0.76	-	0.72	0.24
Weight	0.86	0.33	0.52	0.69	0.79	0.72		0.40
Height	-0.08	-0.02	-0.08	-0.08	0.20	0.24	0.40	-
**Female**								
BMI, current	-	0.36	0.64	0.82	0.78	0.80	0.92	-0.10
BMI, at 18	0.36	-	0.57	0.43	0.22	0.26	0.28	-0.16
BMI, at 35	0.64	0.57	-	0.74	0.48	0.49	0.53	-0.19
BMI, at 50	0.82	0.43	0.74	-	0.66	0.66	0.72	-0.13
Waist	0.78	0.22	0.48	0.66	-	0.78	0.76	0.10
Hip	0.80	0.26	0.49	0.66	0.78	-	0.80	0.17
Weight	0.92	0.28	0.53	0.72	0.76	0.80	-	0.26
Height	-0.10	-0.16	-0.19	-0.13	0.10	0.17	0.26	-

BMI = Body mass index (kg/m^2)
